# Impact of patient positioning on hemodynamic assessment: A comparison of supine and upright right heart catheterization

**DOI:** 10.14814/phy2.70632

**Published:** 2025-10-27

**Authors:** Mohammed A. Chowdhury, Johanna Squires, David M. Systrom, Aaron B. Waxman

**Affiliations:** ^1^ Pulmonary Vascular Disease Program, Brigham and Women's Hospital Boston Massachusetts USA

**Keywords:** right heart catheterization, supine hemodynamics, upright hemodynamics

## Abstract

The impact of patient positioning on PCWP measurement and PH classification is underexplored. This study evaluates the differences in hemodynamic assessments between supine and upright RHC. We conducted a retrospective analysis of a single‐center iCPET registry, comparing sequential hemodynamic measurements in the supine and upright positions in 1307 patients. Compared to upright RHC, supine RHC showed consistently higher mean PAP (21 ± 8.5 mmHg vs. 16 ± 8 mmHg) and PCWP (12 ± 4.7 mmHg vs. 6.4 ± 4.9 mmHg). The differences in mPAP and PCWP measurements between supine and upright RHC persisted regardless of sex, age, BMI, or severity of chronic lung disease. Supine RHC diagnosed significantly more cases of HFpEF (21.8% vs. 5.7%) and isolated postcapillary PH (10.9% vs. 1.8%), primarily due to the overestimation of PCWP. Upright RHC had a higher specificity for detecting elevated PCWP (>15 mmHg) in patients with PCWP/CO>2 (98.27% vs. 84.61%) and demonstrated a stronger association with PCWP/CO>2 (OR 9.11, 95% CI 5–16) compared to supine RHC (*p* = 0.058). PCWP measured in the supine position is higher compared to upright assessment and probably results in increased diagnosis of postcapillary PH.

## 1 INTRODUCTION

Right heart catheterization (RHC) remains the gold standard for hemodynamic assessment and diagnosis of various forms of pulmonary hypertension (PH). Precise measurement of pulmonary artery and pulmonary capillary wedge pressures (PCWP) is critical for distinguishing precapillary from postcapillary PH. Conventionally, RHC is performed in the supine position due to convenient venous access, consistent zeroing of pressure transducers at a standardized anatomical reference point, and minimal variability of posture. In the supine position, there is an increase in ventricular preload due to enhanced venous return and reduced gravitational effects on blood pooling. These changes result in higher filling pressures (Kirupaharan et al., 2024) and cardiac output (Mizumi et al., 2018) potentially mischaracterizing the true hemodynamic status (Berlier et al., 2022; McLaughlin et al., 2009; Mizumi et al., 2018). This limitation is further compounded by patient‐specific factors such as advanced age, obesity, chronic obstructive pulmonary disease, and sleep apnea, which independently affect venous return and intrathoracic pressures (Frank et al., 2020; Frost et al., 2013; Kovacs et al., 2014; Oldenburg et al., 2009). Respirophasic variations during supine RHC can additionally complicate PCWP estimation. Campain et al. demonstrated that the extent of these variations is significantly influenced by age, right atrial pressure, body mass index, and percent predicted FEV1 (Campain et al., 2024). While alternative maneuvers like fluid challenges and exercise RHC have been proposed to improve left‐sided filling pressure evaluation, they predominantly share the inherent limitations of supine positioning (Borlaug et al., 2010; Robbins et al., 2014). Hemodynamic assessment in the upright position offers a potential solution, promising a more physiologically accurate representation of cardiac hemodynamics.

Assessing hemodynamics in the upright position offers several advantages by accurately capturing the body's physiological responses to gravity and posture. Evaluating hemodynamics in the upright position, whether standing or sitting, can offer valuable insights into blood volume and cardiac reserve, particularly in conditions such as heart failure or hypovolemia, where postural changes may exacerbate symptoms. In some cases, significant drops in blood pressure or increases in heart rate during the transition from supine to standing may go undetected if measurements are limited to the supine position. Patients with conditions such as postural tachycardia syndrome (POTS), vasovagal syncope, or preload failure may exhibit hemodynamic abnormalities that are apparent only in the upright position (Joseph et al., 2021, 2023; Singh et al., 2022). In individuals with underlying lung disease or abnormal body habitus, interpreting hemodynamic data requires careful consideration of how these factors influence circulatory and respiratory dynamics when assessed in the supine position (Boerrigter et al., 2014; Campain et al., 2024; Frost et al., 2013; Kovacs et al., 2014; Oldenburg et al., 2009; Smit et al., 2016). Upright hemodynamic assessment enables a more comprehensive understanding of cardiovascular adaptation to gravity, posture, and autonomic regulation. Currently, comprehensive studies comparing hemodynamic assessments in supine versus upright positions are limited, and the extent of variation in these measurements, along with the clinical factors influencing these differences, remains unclear. Adopting a more physiologically representative approach to hemodynamic assessment may enhance diagnostic accuracy, ultimately leading to improved patient management and outcomes. In this study, we evaluated the differences in PCWP estimation between supine and upright RHC and assessed the impact of patient positioning on the classification of precapillary and postcapillary pulmonary hypertension.

## 2 METHOD

### 2.1 Study design

We performed a retrospective, observational analysis comparing the hemodynamics of patients undergoing RHC in both supine and upright positions. The study population was drawn from the Brigham and Women's Hospital Pulmonary Vascular Registry, a comprehensive database of patients who were evaluated for exertional intolerance of unknown etiology with invasive cardiopulmonary exercise testing (iCPET). All patients with an ejection fraction greater than 50% who underwent sequential supine and upright right heart catheterization between January 1, 2015, and January 1, 2024, were included in this analysis. The study was approved by the Institutional Review Board at Brigham and Women's Hospital. Informed consent was obtained from all patients prior to their enrollment in the registry (Huang et al., 2017).

Hemodynamic assessment included measurement of right atrial pressure, right ventricular pressures, pulmonary artery pressure, and PCWP in both supine and upright positions. Cardiac output was estimated by indirect Fick in the supine position, while direct Fick was used to estimate cardiac output in the upright position. Fick‐derived cardiac output was used to calculate pulmonary vascular resistance (PVR) in both supine and upright positions. Additional clinical data, such as demographics, PH classification, and relevant comorbidities, were also recorded.

### 2.2 Right heart catheterization

All patients included in the registry underwent iCPET, which consisted of a resting supine RHC followed by an upright bicycle exercise stress test. A balloon‐tipped, flow‐directed, triple‐lumen, fluid‐filled 7.5 Fr Swan‐Ganz catheter (Baxter/Edwards, Deerfield, IL, USA) was inserted percutaneously under fluoroscopic and ultrasound guidance into the internal jugular vein. Transducers were zeroed in the supine position based on the level of the right atrium, or phlebostatic axis (Rosenkranz & Preston, 2015) and zeroed to atmospheric pressure. The Swan‐Ganz catheter was advanced sequentially through the right atrium, right ventricle, pulmonary artery, and into the right branch of the pulmonary artery to obtain the PCWP, and pressures were measured directly at each location by averaging the hemodynamic pressures over three respiratory cycles. The cardiac output was measured by the indirect Fick method and thermodilution technique. For the Fick method, a blood sample was taken from the pulmonary artery and the radial artery to assess mixed venous and arterial saturation, respectively. Moreover, the thermodilution technique was used to measure cardiac output by injecting a bolus of cold saline into the right atrium via a pulmonary artery catheter. Temperature changes were detected downstream in the pulmonary artery, and cardiac output was calculated based on the area under the thermodilution curve. Following hemodynamic measurements, the pulmonary artery catheter was secured and a sterile dressing was applied. The patients were transported to the stress laboratory in order to undergo a symptom‐limited incremental CPET using an upright cycle ergometer with a breath‐by‐breath assessment of gas exchange (ULTIMA CPX; Medical Graphics Corporation, St Paul, MN, USA). Pulmonary and systemic hemodynamics were continuously and simultaneously monitored during rest and exercise (Xper Cardio Physiomonitoring System; Phillips, Melborne, FL, USA). Subjects were seated upright on the cycle ergometer. The zero reference point for the pressure transducers was identified as 5 cm below the midpoint of the sternum, corresponding to the fourth intercostal space at the midaxillary line, typically about three fingerbreadths below the axilla. The pressure transducer was aligned to this horizontal level to approximate the hydrostatic level of the right atrium. The transducer position was continuously monitored, and adjustments were made if the subject changed position during exercise. An electronic average of hemodynamic pressures over three respiratory cycles was used (Boerrigter et al., 2014).

### 2.3 Diagnostic criteria

The following criteria were used for diagnosis in both supine and upright resting hemodynamic assessment. Precapillary PH was defined as mPAP > 20 mmHg, PCWP ≤ 15 mmHg, and PVR > 2 WU. Combined Pre‐ and Post‐capillary PH (Cpc‐PH) was defined as mPAP > 20 mmHg, PCWP > 15 mmHg, and PVR > 2 WU (Humbert et al., 2022). Isolated Post‐capillary PH (Ipc‐PH) was defined as mPAP > 20 mmHg, PCWP > 15 mmHg, and PVR < 2 WU. Heart failure with preserved ejection fraction (HFpEF) was defined as PCWP > 15 mmHg. iCPET exercise HFpEF was diagnosed using the slope of PCWP/CO with a cutoff of more than 2 (Eisman et al., 2018). iCPET exercise PH was diagnosed using the slope of PAP/CO with a cutoff of more than 3 (Humbert et al., 2022). Preload failure was diagnosed during iCPET using the following criteria; peak VO_2_ < 80% predicted and peak cardiac output <80% predicted and peak right atrial pressure <6.5 mmHg (Oldham et al., 2016). Peripheral limitation which includes mitochondrial myopathy or peripheral left to right shunts was diagnosed during iCPET using the following criteria: peak VO_2_ < 80% and peak cardiac output>80% and peak (CaO_2_‐CVO_2_)/hemoglobin < 80% (Oldham et al., 2016).

### 2.4 Statistical analysis

The variables in the database were assessed for inconsistencies, outliers, and missing data. Outliers were defined as having values more than 1.5 times the interquartile range and were removed to improve the data distribution profile (*n* = 16, 1.2%). Inconsistent or unexpected values were also removed from the database. Missing data (*n* = 28, 2.14%) was imputed by mean or median based on the data distribution of the respective variables. Categorical variables were analyzed using Pearson's Chi‐Square test, while continuous variables were assessed using either the Kruskal–Wallis test or *T*‐test, depending on the distribution of the data. For multiple comparisons, *p* values were corrected using the Benjamini–Hochberg procedure to control the false discovery rate (FDR). A significance threshold of FDR‐adjusted *p* < 0.05 was used. To evaluate the diagnostic performance of PCWP measurements in the supine and upright positions relative to a validated diagnostic modality for HFpEF, we used binary logistic regression to assess the association between a PCWP greater than 15 mmHg in both positions and iCPET‐diagnosed HFpEF.

## 3 RESULTS

A total of 1307 patients were analyzed, with a mean age of 56 ± 16 years, including 66.2% females (*n* = 864) and 33.8% males (*n* = 443), with a mean body mass index (BMI) of 28.7 ± 7 kg/m^2^. Additional cohort characteristics have been listed in Table 1.

**TABLE 1:** Study cohort demographics.

Study cohort demographics	
Age (mean ± SD)	55.4 ± 17.6
Sex: male (*n*, %)	933, (71.4%)
BMI (mean ± SD)	28.7 ± 11.8

Established medical history	
Group 1 pulmonary hypertension (*n*, %)	87, (7%)
Group 2 pulmonary hypertension (*n*, %)	103, (8%)
Group 3 pulmonary hypertension (*n*, %)	44, (3%)
Group 4 pulmonary hypertension (*n*, %)	9, (6.5%)
Group 5 pulmonary hypertension (*n*, %)	11, (0.8%)
Exercise pulmonary hypertension (*n*, %)	193, (15%)
Coronary artery disease (*n*, %)	55, (4%)
Cerebrovascular accident (*n*, %)	6, (0.4%)
Myocardial infarction (*n*, %)	15, (1.1%)
Peripheral arterial disease (*n*, %)	4, (0.3%)
Valvular heart disease (*n*, %)	49, (3.7%)
Ever a smoker (*n*, %)	517, (39%)
H/o drug use (*n*, %)	30, (2%)
Obesity (*n*, %)	14, (1%)
Cancer (*n*, %)	82, (6%)
Type I diabetes mellitus (*n*, %)	7, (0.5%)
Type II diabetes mellitus (*n*, %)	99, (7.5%)
Hypertension (*n*, %)	227, (17%)
HIV (*n*, %)	3, (0.2%)
Deep venous thrombosis (*n*, %)	32, (2%)
Pulmonary embolism (*n*, %)	42, (3.2%)
Hypothyroidism (*n*, %)	95, (7%)
Hyperthyroidism (*n*, %)	5, (0.3%)
Chronic kidney disease (*n*, %)	27, (2%)

Presenting symptoms	
Dyspnea at rest (*n*, %)	25, (1.9%)
Fatigue (*n*, %)	221, (17%)
Chest pain/discomfort (*n*, %)	109, (8%)
Cough (*n*, %)	88, (7%)
Palpitations (*n*, %)	42, (3%)
Orthopnea (*n*, %)	39, (2.9%)
Presyncope/syncope (*n*, %)	43, (3.2%)
Edema (*n*, %)	61, (4.6%)
Raynaud's phenomenon/disease (*n*, %)	22, (1.6%)
Anorexia (*n*, %)	4, (0.3%)
Atrial arrythmia (*n*, %)	10, (0.7%)
Paroxysmal nocturnal dyspnea (*n*, %)	7, (0.5%)
Dizziness/lightheadedness (*n*, %)	133, (10.1%)

Medications	
Calcium channel blockers (*n*, %)	139, (10.6%)
Long‐acting nitrates (*n*, %)	29, (2%)
ACE inhibitors/Angiotensin receptor blocker (*n*, %)	211, (16%)
Beta blockers (*n*, %)	279, (21%)
Corticosteroids (*n*, %)	316, (24%)
Aspirin (*n*, %)	272, (20%)
NSAID (*n*, %)	254, (19%)
Clopidogrel (*n*, %)	28, (2%)
Thiazide (*n*, %)	64, (4.9%)
Loop diuretic (*n*, %)	188, (14%)
Spironolactone (*n*, %)	50, (3.8%)
Digoxin (*n*, %)	13, (1%)
Statin (*n*, %)	302, (23%)
Selective serotonin reuptake inhibitor (*n*, %)	232, (17%)

Laboratory data	
Hemoglobin g/L (mean ± SD)	14 ± 2
Creatinine mg/dL (median, IQR)	0.89 (0.3)
BUN mg/dL (mean ± SD)	17 ± 12.5
NT‐proBNP pg/mL (median, IQR)	140 (528)

Resting supine hemodynamics	
Systolic blood pressure mmHg (mean ± SD)	142 ± 60
Diastolic blood pressure mmHg (mean ± SD)	81 ± 26
Mean right atrial pressure mmHg (mean ± SD)	6 ± 3.6
Right ventricular peak systolic pressure mmHg (mean ± SD)	31 ± 12
Right ventricular end diastolic pressure mmHg (mean ± SD)	8 ± 4
Systolic pulmonary artery pressure mmHg (mean ± SD)	30 ± 14
Diastolic pulmonary artery pressure mmHg (mean ± SD)	13 ± 6
Mean pulmonary artery pressure mmHg (mean ± SD)	20 ± 9
Mean PCWP pressure mmHg (mean ± SD)	11.5 ± 5.4
Fick cardiac output L/min (mean ± SD)	5.3 ± 1.2
Fick cardiac index L/min/m^2^ (mean ± SD)	2.8 ± 0.8
Thermodilution cardiac output L/min (mean ± SD)	5.2 ± 1.2
Thermodilution cardiac index L/min/m^2^ (mean ± SD)	2.7 ± 0.5
Pulmonary vascular resistance dynes/sec/cm‐5 (mean ± SD)	152 ± 134

Resting upright hemodynamics	
Systolic blood pressure mmHg (mean ± SD)	145 ± 24
Diastolic blood pressure mmHg (mean ± SD)	79 ± 12
Mean right atrial pressure mmHg (median, IQR)	2 (4)
Mean pulmonary artery pressure mmHg (mean ± SD)	14 ± 7
Mean PCWP pressure mmHg (mean ± SD)	6 ± 4
Fick cardiac output L/min (mean ± SD)	5.67 ± 1.9
Pulmonary vascular resistance dynes/sec/cm‐5 (mean ± SD)	152 ± 114

Diagnosis after invasive cardiopulmonary stress test	
Exercise HFpEF (*n*, %)	145, (11.1%)
Exercise pulmonary hypertension (*n*, %)	278, (21.3%)
Preload failure (*n*, %)	492, (37.6%)
Peripheral limitation (*n*, %)	179, (13.7%)
Normal iCPET study (*n*, %)	213, (16.3%)

### 3.1 Hemodynamic measurements

#### 3.1.1 Supine versus upright right heart catheterization

Supine RHC recorded an average mean pulmonary artery pressure (mPAP) of 21 ± 8.5 mmHg, compared to 16 ± 8 mmHg in the upright position, with a mean difference of 5 ± 5.4 mmHg (*p* < 0.0001). In the supine position, 42% of patients were classified as having mPAP >20 mmHg, versus 21% in the upright position. The average pulmonary capillary wedge pressure (PCWP) was 12 ± 4.7 mmHg supine and 6.4 ± 4.9 mmHg upright, showing a significant mean difference of 5.7 ± 4.6 mmHg (*p* < 0.0001). Supine RHC indicated PCWP >15 mmHg in 21.8% of patients, compared to only 5.67% in the upright position. There was no significant difference in pulmonary vascular resistance between the supine and upright positions. The agreement rate between supine and upright measurement for mPAP >20 mmHg and PCWP >15 mmHg was 82% and 28%, respectively.

#### 3.1.2 Pulmonary hypertension classification

**FIGURE 1:**
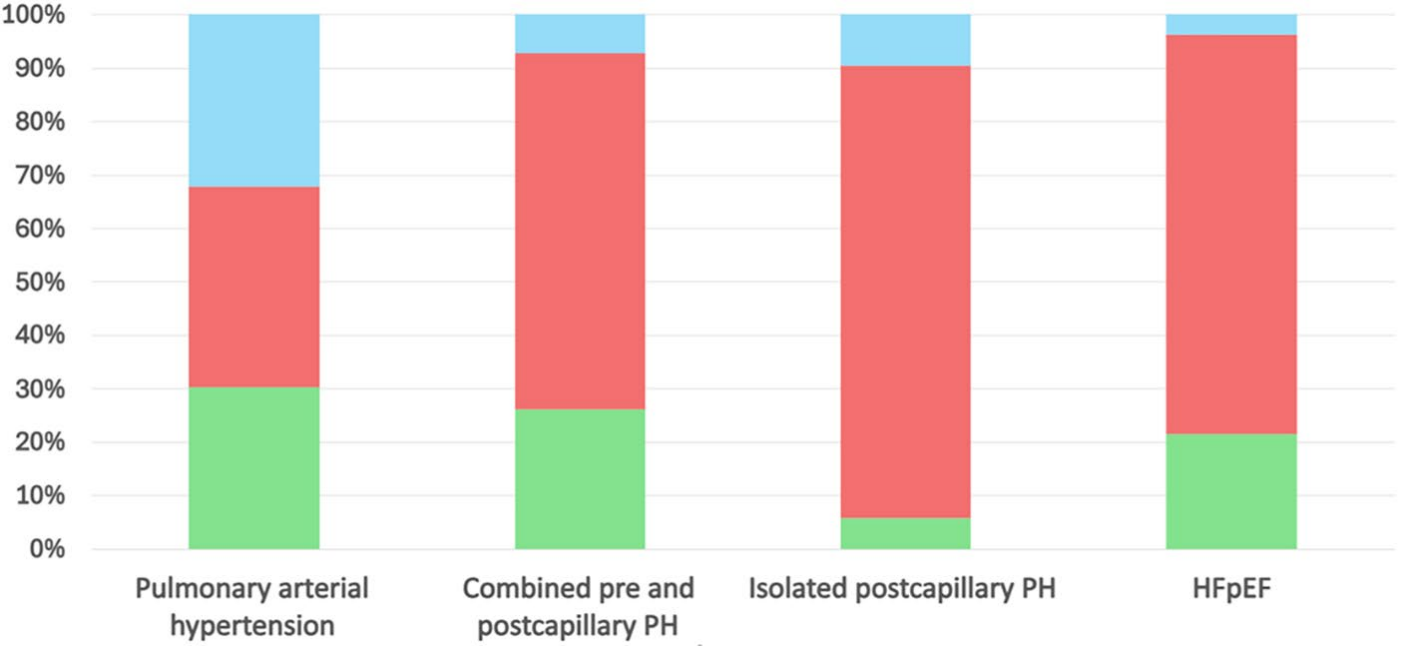
Bar graph illustrating the distribution of PH and HFpEF diagnoses stratified by RHC performed in the supine position only (red), upright position only (blue), and by both modalities (green). The proportions reflect the relative contribution of each testing position to the overall identification of PH and HFpEF within the study cohort.

Precapillary PH was diagnosed in 13.4% of patients using RHC and in 12.3% using upright RHC, indicating similar diagnostic rates between the two positions. In contrast, Cpc‐PH was identified in 9.8% of patients in the supine position compared to 3.52% in the upright position. Ipc‐PH was found in 10.87% of supine RHC versus 1.84% upright RHC. HFpEF was diagnosed in 21.75% of cases using supine RHC, compared to 5.67% with upright RHC. These findings suggest that supine RHC generally results in higher PCWP measurements, leading to more frequent classification of HFpEF, Cpc‐PH, and Ipc‐PH (Table 2 and Figure 1).

**TABLE 2:** Differences in hemodynamic measurement and PH classification between supine and upright RHC.

	Supine RHC	Upright RHC	Difference between supine and upright measurement	*p* Value
Right atrial pressure (mmHg)	6.8 ± 3.5	2.7 ± 3.5	4.1 ± 3.8	**<0.0001**
Mean pulmonary artery pressure (mmHg)	21 ± 8.5	16 ± 8	5 ± 5.4	**<0.0001**
Pulmonary capillary wedge pressure (mmHg)	12 ± 4.7	6.4 ± 4.9	5.7 ± 4.6	**<0.0001**
Pulmonary vascular resistance (WU)	1.88 ± 1.6	1.9 ± 1.4	−0.04 ± 1.3	0.2
Fick cardiac output (L/min)	5.3 ± 1.2	5.6 ± 1.8	−0.3 ± 2	**<0.0001**
mPAP>20 mmHg (*n*, %)	42% (*n* = 549)	21% (*n* = 275)	21%	**<0.0001**
PCWP>15 mmHg (*n*, %)	21.8% (*n* = 284)	5.67% (*n* = 74)	16.13%	**<0.0001**
Pulmonary arterial hypertension (*n*, %)	13.4% (*n* = 175)	12.3% (*n* = 161)	1.1%	**<0.0001**
Combined pre and post capillary PH (*n*, %)	9.8% (*n* = 128)	3.52% (*n* = 46)	6.28%	**<0.0001**
Isolated post capillary PH (*n*, %)	10.87% (*n* = 142)	1.84% (*n* = 24)	9.03%	**<0.0001**

*Note*: Bold format reflect the values that are statistically significant.

#### 3.1.3 Influence of physiological factors

Males exhibited higher mPAP and PCWP than females in both positions, with supine measurements being consistently higher. Both mPAP and PCWP increased with advancing age, increasing BMI, and worsening lung disease (Table 3), with supine values consistently higher than upright. Patients with a history of smoking or CPAP/BiPAP use showed varied hemodynamic responses between positions, affecting mPAP less than PCWP (Table 4). In our registry, preload failure was diagnosed in 37.6% of patients (*n* = 492). Among these individuals, the difference in PCWP between supine and upright positions persisted, with a mean difference of 3.7 ± 0.2 mmHg.

**TABLE 3:** Differences in hemodynamic measurement between supine and upright RHC according to age, BMI, and FEV1. *p* Values were corrected for multiple comparisons using the Benjamini–Hochberg procedure (False Discovery Rate control at *α* = 0.05).

Age	Supine RHC	Upright RHC	Difference	*p* Value	Bonferroni adjusted *p* value	FDR adjusted *p* value
mPAP						
<65 years (*n* = 840)	18.2 ± 7.2	13.8 ± 6.5	4.4 ± −0.3	<0.0001	0.003	0.0002
>65 years (*n* = 467)	24.85 ± 10	20 ± 9.6	4.9 ± 0.6	<0.0001	0.003	0.0002
PCWP						
<65 years (*n* = 840)	10.6 ± 4.6	5 ± 4	5.6 ± 0.2	<0.0001	0.003	0.0002
>65 years (*n* = 467)	13 ± 6	8.5 ± 5.6	4.5 ± 0.4	<0.0001	0.003	0.0002
PVR						
<65 years (*n* = 840)	1.5 ± 1.3	1.6 ± 1	−0.1 ± 0.05	0.07	1.0	0.0955
>65 years (*n* = 467)	2.5 ± 1.9	2.47 ± 1.8	−0.03 ± 0.13	0.8	1.0	0.81

BMI (Kg/m^2^)	Supine RHC	Upright RHC	Difference	*p* Value	Bonferroni adjusted *p* value	FDR adjusted *p* value
mPAP						
<25 (*n* = 470)	17.2 ± 7.5	12.5 ± 7	4.7 ± 0.49	**<0.0001**	**0.003**	**0.0002**
25–30 (*n* = 360)	19.8 ± 8.7	15.8 ± 8.5	4 ± 0.66	<0.0001	0.003	0.0002
31–35 (*n* = 306)	22 ± 8.3	17.7 ± 8.1	4.3 ± 0.7	**<0.0001**	**0.003**	**0.0002**
36–40 (*n* = 106)	23.6 ± 8.1	18.5 ± 6.9	5.1 ± 0.96	**<0.0001**	**0.003**	**0.0002**
>41 (*n* = 65)	24.8 ± 9.6	20.8 ± 8.5	4 ± 1.4	**<0.0001**	**0.003**	**0.0002**
PCWP						
<25 (*n* = 470)	9.95 ± 4	3.7 ± 3.6	6.25 ± 0.3	**<0.0001**	**0.003**	**0.0002**
25–30 (*n* = 360)	10.9 ± 5	6.3 ± 5.1	4.6 ± 0.39	**<0.0001**	**0.003**	**0.0002**
31–35 (*n* = 306)	11.9 ± 5.4	7.2 ± 4.5	4.7 ± 0.44	**<0.0001**	**0.003**	**0.0002**
36–40 (*n* = 106)	13.8 ± 5.9	8.5 ± 4.9	5.3 ± 0.69	**<0.0001**	**0.003**	**0.0002**
>41 (*n* = 65)	13.8 ± 6.1	9.5 ± 4.4	4.3 ± 0.8	**<0.0001**	**0.003**	**0.0002**
PVR						
<25 (*n* = 470)	1.6 ± 1.6	1.8 ± 1.4	−0.2 ± 0.1	**0.05**	1.0	0.0714
25–30 (*n* = 360)	1.9 ± 1.6	2 ± 1.8	−0.1 ± 0.1	0.4	1.0	0.4615
31–35 (*n* = 306)	1.99 ± 1.59	1.9 ± 1.2	0.09 ± 0.1	0.5	1.0	0.5556
36–40 (*n* = 106)	1.93 ± 1.15	1.76 ± 1.2	0.17 ± 0.15	0.3	1.0	0.375
>41 (*n* = 65)	2 ± 1.3	1.8 ± 1.2	0.2 ± 0.2	0.3	1.0	0.375

FEV1	Supine RHC	Upright RHC	Difference	*p* Value	Bonferroni adjusted *p* value	FDR adjusted *p* value
mPAP						
FEV1 > 80% (*n* = 623)	18.8 ± 7	14 ± 5.6	4.8 ± 0.3	**<0.0001**	**0.003**	**0.0002**
FEV1 50%–80% (*n* = 312)	24 ± 10	20 ± 10	4 ± 0.8	**<0.0001**	**0.003**	**0.0002**
FEV1 < 50% (*n* = 372)	31 ± 10.5	26 ± 9.5	5 ± 2	**0.01**	0.3	**0.015**
PCWP						
FEV1 > 80% (*n* = 623)	10.9 ± 4.5	5.7 ± 4	5.2 ± 0.2	**<0.0001**	**0.003**	**0.0002**
FEV1 50–80% (*n* = 312)	12.5 ± 6	8 ± 5.2	4.5 ± 0.46	**<0.0001**	**0.003**	**0.0002**
FEV1 < 50% (*n* = 372)	15.2 ± 6.7	11.5 ± 6.9	3.7 ± 1.3	**<0.006**	0.18	**0.0095**
PVR						
FEV1 > 80% (*n* = 623)	1.6 ± 1.25	1.69 ± 1.17	0.09 ± 0.07	0.21	1.0	0.27
FEV1 50%–80% (*n* = 312)	2.46 ± 2.14	2.5 ± 1.97	0.04 ± 0.16	0.81	1.0	0.81
FEV1 < 50% (*n* = 372)	3.3 ± 2.2	3 ± 1.6	0.3 ± 0.3	0.4	1.0	0.44

*Note*: Bold format reflect the values that are statistically significant.

**TABLE 4:** Influence of miscellaneous factor on mPAP, PCWP and PVR stratified according to supine and upright RHC measurement.

	Supine RHC	Upright RHC	Difference in measurement	*p* Value
mPAP				
Nonsmoker (*n* = 790)	18.8 ± 9.7	14.3 ± 6.7	4.5 ± 0.4	**<0.0001**
Smoker (*n* = 517)	23 ± 9.6	18.5 ± 9.8	4.5 ± 0.6	**0.0004**
Not on home oxygen (*n* = 1282)	19.7 ± 8.2	15 ± 7.6	4.7 ± 0.4	**<0.0001**
On home oxygen (*n* = 25)	27.8 ± 12	25 ± 9.6	2.8 ± 0.3	0.4
No CPAP/BiPAP use (*n* = 1222)	20 ± 8.5	15 ± 7.8	5 ± 0.4	**<0.0001**
CPAP/BiPAP use (*n* = 85)	19 ± 17	15 ± 13.5	4 ± 2.5	0.1
Not on loop diuretics (*n* = 1119)	17.8 ± 6.5	13.3 ± 5.8	4.5 ± 0.3	**<0.0001**
On loop diuretics (*n* = 188)	28.8 ± 10.7	23.2 ± 9.7	5.6 ± 1.7	**0.001**
No preload failure (*n* = 815)	21.9 ± 9.6	15.98 ± 8.2	5.9 ± 0.5	**<0.0001**
Preload failure (*n* = 492)	16.7 ± 5.27	16.4 ± 8.3	0.3 ± 0.4	0.5
PCWP				
Nonsmoker (*n* = 790)	10.8 ± 4.7	5.3 ± 4.3	5.5 ± 0.2	**<0.0001**
Smoker (*n* = 517)	12.5 ± 5.8	7.6 ± 5.5	4.9 ± 0.4	**<0.0001**
Not on home oxygen (*n* = 1282)	11.2 ± 5	5.7 ± 4.8	5.5 ± 0.3	**<0.0001**
On home oxygen (*n* = 25)	12.7 ± 9.5	10.37 ± 6.6	2.3 ± 2.3	0.3
No CPAP/BiPAP use (*n* = 1222)	11.2 ± 5	5.8 ± 4.8	5.4 ± 0.2	**<0.0001**
CPAP/BiPAP use (*n* = 85)	11.3 ± 9.9	6.2 ± 5.5	5.1 ± 1.3	**0.0002**
Not on loop diuretics (*n* = 1119)	10.3 ± 4	4.7 ± 3.8	5.6 ± 0.2	**<0.0001**
On loop diuretics (*n* = 188)	15.3 ± 14	10.7 ± 9.8	4.6 ± 1.3	**0.0005**
No preload failure (*n* = 815)	12 ± 5.56	6.4 ± 5	5.6 ± 0.2	**<0.0001**
Preload failure (*n* = 492)	10 ± 3.95	6.3 ± 4.87	3.7 ± 0.2	**<0.0001**

*Note*: Bold format reflect the values that are statistically significant.

### 3.2 Diagnostic accuracy of supine versus upright RHC in assessing left‐sided filling pressures

To compare the diagnostic performance of supine and upright RHC in detecting elevated left‐sided filling pressures, we analyzed the association of PCWP >15 mmHg measured in each position with iCPET‐diagnosed exercise HFpEF, defined by an incremental exercise PCWP/CO slope >2. Supine RHC achieved a sensitivity of 55.29% and specificity of 84.61%, compared to a sensitivity of 26.44% and specificity of 98.27% for upright RHC. Binary logistic regression analysis revealed that elevated PCWP measured by upright RHC (OR 9.11, 95% CI 5–16, *p* < 0.0001) had a stronger association with PCWP/CO >2 compared to supine RHC (OR 4.4, 95% CI 3–6, *p* < 0.0001). We compared the strength of association between elevated PCWP and PCWP/CO >2 measured by upright versus supine RHC within the same logistic regression model using a contrast test. Although the odds ratio for upright PCWP was numerically higher than for supine PCWP, the difference did not reach statistical significance (Wald χ^2^ = 3.58, *p* = 0.058).

## 4 DISCUSSION

Our study reveals a significant discrepancy in the measurement of mPAP and PCWP between supine and upright RHC. Specifically, supine RHC tends to measure higher mPAP and PCWP compared to upright RHC. This discrepancy has potential critical implications for diagnosing various forms of PH, including precapillary PH, Cpc‐PH, Ipc‐PH, and HFpEF. Moreover, it may misrepresent the hemodynamic changes that occur during upright exertion. Accurate diagnosis of PH relies on precise measurements of mPAP, PCWP, and PVR (Joseph et al., 2021). Current diagnostic criteria, such as mPAP >20 mmHg and PCWP <15 mmHg, are predominantly based on supine RHC measurements. This raises the possibility that some patients with precapillary PH could be misclassified as having Cpc‐PH, which may influence treatment decisions. Accurate classification is crucial because management strategies differ significantly. PAH therapies focus on the pulmonary vasculature, while Cpc‐PH management addresses underlying left heart disease (Smit et al., 2016).

In a similar study of 36 consecutive patients who first underwent submaximal supine exercise at 20 watts, followed by a 20–25 minute rest period and then a maximal upright exercise test, Fudim et al. reported that 50% of the cohort had discordant HFpEF diagnoses between the supine and upright exercise tests (Fudim et al., 2024). The authors concluded that hemodynamic measurements obtained during supine exercise do not necessarily reflect the physiological conditions experienced during upright physical activity. Similarly, other papers have also questioned PCWP measurement in the supine position (Rayner et al., 2024). This aligns with our findings, which demonstrate that PCWP measurements are affected by body position. Additionally, the difference in resting PCWP between supine and upright positions in the above‐mentioned study was 5 ± 9 mmHg, which closely matches our observed mean difference of 5 mmHg, further supporting the generalizability of our findings. Similarly, Kirupaharan et al. demonstrated similar significant differences in PCWP measurements between supine and upright positions across all stages of exercise testing (Kirupaharan et al., 2024). Both studies emphasized that supine exercise testing, due to increased venous return, may mask preload failure, thereby limiting the ability to diagnose this important HFpEF phenotype. This has clinical implications since patients with preload failure are often intolerant to vasodilators and aggressive diuresis.

Upright positioning is preferred for hemodynamic assessment using a pulmonary artery line in patient populations undergoing exercise hemodynamics, particularly those being evaluated for PH or exercise‐PH (Berlier et al., 2022). In these patients, upright measurements more closely reflect physiologic conditions encountered during daily activities and exercise and can unmask abnormalities not evident in the supine position (Hsu et al., 2022). Raina et al. compared pulmonary pressures between baseline RHC and implantable hemodynamic monitors and reported that 48.8% of patients were relabeled as pulmonary hypertension based on their ambulatory hemodynamics, which was not previously detected on resting supine RHC, underscoring the limitations of our current practice (Raina et al., 2015). Upright assessment also reveals variability in PCWP and other hemodynamic parameters that may be obscured in the supine position, which is important for diagnosing PH and evaluating cardiac reserve under physiological stress (Jain & Borlaug, 2020). Supine RHC is further limited by pericardial restraint, which can cause right ventricular distension at the expense of left ventricular filling during preload increases (Moore et al., 2001). Moreover, obesity usually elevates intra‐abdominal pressure in the supine position thus increasing venous return and falsely raising cardiac filling pressures (Smit et al., 2016). Our findings emphasize the importance of incorporating upright hemodynamic assessment into clinical practice to enhance diagnostic accuracy and guide appropriate management of PH subtypes.

The PCWP cutoff of 15 mmHg has been well validated in the supine position (Binanay et al., 2005); however, no equivalent cutoff has been established for the upright position. Previously, our group reported the average cutoff for PCWP in the upright position at rest to be 12 ± 4 mmHg in patients ≤50 years and 12 ± 3 mmHg in patients >50 years (Oliveira et al., 2016). In this study, our analysis demonstrated that applying the same 15 mmHg threshold to upright PCWP measurements results in decreased sensitivity but increased specificity for detecting elevated left‐sided filling pressures. This analysis was intended solely to illustrate the differences in PCWP measurements between supine and upright positions relative to iCPET diagnosed HFpEF. Further research is needed to determine an appropriate PCWP cutoff for the resting upright position. Nonetheless, our findings indicate that upright PCWP measurement offers greater specificity compared to supine measurements. The high specificity of upright RHC is particularly beneficial when evaluating patients suspected of having PH, as it reduces the likelihood of false positives in diagnosing elevated left‐sided filling pressures and prevents the misclassification of patients with pulmonary arterial hypertension as having Cpc‐PH. Consequently, this approach ensures that patients receive the correct diagnosis and appropriate care.

In our cohort, the mPAP and PCWP increased with advancing age, higher BMI, lower FEV1, and were also influenced by sex, diuretic use, smoking history, and the diagnosis of preload failure. However, the differences between supine and upright measurements persisted despite the presence of the above‐mentioned factors (Table 4). Interestingly, pressures were higher in patients receiving diuretics, likely reflecting greater volume status and elevated filling pressures; however, the positional differences remained consistent regardless of diuretic use.

To the best of our knowledge, our study includes the largest sample of patients to date with sequential supine and upright hemodynamic measurements. Nonetheless, our study has several limitations. This is a retrospective study, and the observed results could be influenced by selection bias and unmeasured confounding variables. However, it is important to note that although the data analysis was conducted retrospectively, the data collection was prospective. Specifically, all patients were systematically scheduled to undergo a resting RHC followed by an upright hemodynamic assessment. The single‐center nature of the research may limit the generalizability of the findings to other populations due to potential demographic homogeneity within the sample. Moreover, our study spanned a period of 9 years, during which evolving referral patterns for the evaluation of dyspnea of unknown etiology to our dyspnea clinic may have contributed to the slight variability observed in our results. In addition, cardiac output was estimated using the indirect Fick method in the supine position, whereas the direct Fick method was used in the upright position. This difference in measurement technique may have contributed to the higher cardiac output and PVR observed in our study cohort. Although our overall study sample was large, the number of HFpEF patients diagnosed using supine RHC and iCPET was relatively small, representing another limitation of this study.

Our findings would suggest that it is time to consider an alternative approach to the evaluation of patients with PH. Further studies are warranted to explore the feasibility, benefits, and outcomes of these proposed strategies.

## AUTHOR CONTRIBUTIONS

All authors contributed equally to the research and preparation of the manuscript.

## FUNDING INFORMATION

None.

## CONFLICT OF INTEREST STATEMENT

None.

## ETHICS STATEMENT

Approved by IRB of Brigham and Women's Hospital.

## Data Availability

The data that support the findings of this study are available on request from the corresponding author. The data are not publicly available due to privacy or ethical restrictions.
